# The Story of the Insane from Year to Year

**Published:** 1892-04-09

**Authors:** 


### The Story of the Insane from Year to Year.

(Fourth Series.)

### GLOUCESTER COUNTY ASYLUMS.

The numbers rose from 987 to 1,014, showing an increase of 27 during the year. There were 292 admissions, 105 recoveries and 122 deaths. Put into percentages the recoveries come out at 43·75 and the deaths at 12·27, both of these being above the average. Twenty-two of the admissions owed their insanity to hereditary influences and the same number to drink. Various brain diseases account for 35 of the deaths and phthisis for 14. Dr. Craddock tells us how discouraging it is to note the unfavourable nature of the admissions during 1890, many having their chances of recovery "reduced to a vanishing point," either by reason of bad general health or of the duration of the insanity previous to admission, and he thinks the new Lunacy Act has interposed new obstacles in way of early treatment. Dr. Craddock further says that the first year's experience of the Act has not increased his regard "either for the intrinsic merits of this meretricious piece of legislation itself, nor the tact and acumen with which its provisions have been interpreted by those whose business it is to, presumably, administer such Acts of Parliament. The minute scrutiny of detail which rejects a reception order because the number of John Smith's house is omitted, and the eagerness for information, which is betrayed by the desire to know whether a man reported to be suffering from mania stands on his head or not, seem hardly compatible with the negligence which fails to ascertain that the plans for a large and costly building for which the ratepayers will have to pay many thousands of pounds, are of such a character as to be suitable for the care and safety of the patients for whom they are designed." The Gloucester Committee of Visitors have decided not to admit any more private patients after the New Lunancy Act came into operation. No one can be surprised at this step, but Dr. Craddock himself recognizes the hardship which the step will entail on many poor inhabitants of the county. Would it not be better to have received the patients as usual, for a year or two at all events, and in the meantime have tried to get this ridiculous Act wiped off the Statute Book? Dr. Craddock falls foul of the Government audit. For this "second performance the county has the pleasure of paying about £20 a-year." We believe we could, in conjunction with Dr. Craddock, spend the £20 to much better advantage. Poor John Bull does not seem able to exist without periodical panics! Sometimes it is a mortal dread lest the French should land at Dover; or the Germans fight the battle of Dorking; or the Russians force the Kyber Pass. Lately it was the condition of lunatic asylums, which of all our institutions were in the most satisfactory state, and least needed tinkering. The visitors say, "The asylums have been maintained in perfectly good order under the able management of Mr. Craddock, who justly enjoys the full confidence of the Committee." The weekly rate is 7s. 9d.

### WARWICK COUNTY ASYLUM.

In this Asylum there was during the year 1890 a decrease in the numbers. The year began with 659, and ended with 645. There were 141 admissions, 61 recoveries, and 78 deaths. The percentages are respectively 43·2 and 12. Of the admissions, we find that 48 were owing to hereditary influence and 12 to drink. Thirty of the deaths were caused by brain diseases and eight by phthisis. Dr. Nicholson, the assistant medical officer, is following the example set in many asylums, and delivering lectures on "first aid" and ambulance work. Dr. Miller says that already on more than one occasion the knowledge so obtained by the attendants has proved of signal service. The Warwick Asylum is full to overflowing, and sixty patients have been boarded out in other asylums. Dr. Miller tells us the New Lunacy Act has been the means of increasing the office work to a very considerable degree, and a Superintendent's clerk has been appointed. This officer, among other things, "enters nearly all the notes in the case books, which have then only to be signed by the medical staff." We would point out that, however desirable this arrangement may be, it is contrary to the new rules, which requires that the entries shall be signed by the person making them. The weekly rate is 9s. 4d.

### DURHAM COUNTY ASYLUM.

On December 31st, 1889, this asylum contained 1,089 patients, and on the same day of 1890 the number had risen to 1,171. The admissions were 301; the recoveries 147, and the deaths 159. These yield the following percentages: recoveries, 36·6 on the admissions; and deaths, 18·9 on the average number resident. Thirty of the deaths were owing to phthisis, and 52 to brain diseases. Dr. Smith puts the New Lunacy Act in a nutshell. He christens it, "An Act to prevent the early treatment of the insane, and to hamper all having the care of them." The weekly rate of maintenance is 9s.

### BERKS COUNTY ASYLUM.

The patients on January 1st, 1890, numbered 502; and on December 31st they had reached 514. The admissions were 99, the recoveries 31, and the deaths 40. The percentages, therefore, stood at 31·3 recoveries, and 7·7 deaths. Hereditary influence accounted for 14 of the admissions, and intemperance in drink for 5. The recovery rate is below the average; but it can hardly be wondered at, inasmuch as the previous year showed a very high percentage, and as 65 per cent. of the year's admissions were chronic cases, and only 7 of the total admissions were in good health. When speaking of suicidal patients, and of the anxiety such cases cause to the staff, Dr. Donty refers to the usual custom of giving the attendants "caution cards" with each suicidal case, and he says he takes "the responsibility of withdrawing suicidal caution tickets as soon as possible after admission of each case, as appears to me to be possible;" and he gives as his reason for this proceeding, that familiarity with large numbers of suicidal cases produces contempt for their individual importance. There is something to be said for this view. Dr. Donty criticises the Lunacy Act at some length, and we extract the following from his remarks. "I confess that, after meditating largely upon them during the last twelve months, and trying to unravel the mysteries of the restrictions and stipulations under which these returns are to be made, I cannot believe a single member of Parliament would have helped to pass in committee the sections relating to these reports, could he have spent prospectively an hour in a Medical Superintendent's Office, and have had explained to him there, the absurd complications and waste of time which must accompany the working of these sections. Repeal is, one would think, a necessary consequence in the near future." This journal was among the first to point out the absurdities of the Act, and we are pleased to find that those who have to administer it agreed with the opinions we long since expressed. The weekly rate of maintenance is 8s.

